# Community Based Case-Control Study of Rotavirus Gastroenteritis among Young Children during 2008-2010 Reveals Vast Genetic Diversity and Increased Prevalence of G9 Strains in Kolkata

**DOI:** 10.1371/journal.pone.0112970

**Published:** 2014-11-17

**Authors:** Satarupa Mullick, Anupam Mukherjee, Santanu Ghosh, Gururaja P. Pazhani, Dipika Sur, Byomkesh Manna, James P. Nataro, Myron M. Levine, Thandavarayan Ramamurthy, Mamta Chawla-Sarkar

**Affiliations:** 1 National Institute of Cholera and Enteric Diseases, Kolkata, India; 2 Department of Paediatrics, University of Virginia, School of Medicine, Charlottesville, Virginia, United States of America; 3 Center for Vaccine Development, University of Maryland, School of Medicine, Baltimore, Maryland, United States of America; Instituto de Higiene e Medicina Tropical, Portugal

## Abstract

**Background:**

Group A Rotaviruses are a major etiologic agent of gastroenteritis in infants and young children (<5 years) worldwide. Although rotavirus vaccines have been successfully administered in many countries, in India the introduction of rotavirus vaccine in national immunization program was approved in 2014. Since high disease burden and large number of genetic variants have been reported from low income countries including India, monitoring of rotavirus was initiated prior to implementation of the vaccine in the region.

**Methods:**

A total number of 3,582 stool samples were collected from an urban slum community in Kolkata, among which 1,568 samples were obtained from children of ≤5 years of age, with moderate to severe diarrhoea and 2,014 samples were collected from age-sex matched healthy neighbourhood controls. Rotavirus positive samples were typed by multiplex semi-nested PCR and nucleotide sequencing. Circulating strains were phylogenetically analyzed.

**Results:**

Among 1,568 children with diarrhoea, 395 (25.2%), and among 2,014 asymptomatic children, 42 (2%) were rotavirus positive. G1P[8] was identified as the most common strain (32%) followed by G9P[8] (16.9%), G2P[4] (13.5%) and G9P[4] (10.75%). G12 strains with combinations of P[4], P[6] and P[8] comprised 11.9% of total positive strains. The rest (<10%) were rare and uncommon strains like G1P[4], G1P[6], G2P[8] and animal-like strains G4P[6], G6P[14] and G11P[25]. The 42 rotavirus positive samples from asymptomatic children revealed common genotypes like G1, G2 and G9.

**Conclusion:**

This community based case-control study showed increased predominance of genotype G9 in Kolkata. It also confirmed co-circulation of a large number of genetic variants in the community. Asymptomatic rotavirus positive children though low in number can also be a source of dispersal of infection in the community. This study provides background information to the policy makers for implementation of rotavirus vaccines in this region.

## Introduction

Severe gastroenteritis in children below 5 years of age is a major public health problem in humans globally [1]. Worldwide approximately 10.6 million children die before their fifth birthday of which 20% deaths are attributed to diarrhoeal diseases [2]. Although large number of bacterial, viral and parasitic pathogens have been implicated to cause diarrhoea, but Group A rotavirus (RVA) has been identified to cause severe diarrhoea and approximately 453,000 deaths among children <5 years of age [3]–[8]. A three year prospective, age stratified, matched case-control study (Global Enteric Multicentre Study or GEMS) was conducted in censused population at four sites in Africa and three in Asia [9] to identify pathogen specific paediatric diarrhoeal disease burden in children aged <5 years. GEMS Study confirmed RVA as the most common pathogens in 0–2 year’s age group in sub-Saharan Africa and South Asia [9], [57].

The RVA belong to family Reoviridae with a double stranded RNA genome contributing 11 gene segments. Due to segmented genome, genetic reassortments are common among co-circulating strains of human or animal origin [10]. This results in generation of large number of genetic variants for example, currently 27G, 37P, 16I, 9R, 9C, 8M, 16A, 9N, 12T, 14E and 11H genotypes have been identified [11], [12]. Six RVA genotypes namely G1, G2, G3, G4, G9 and G12 in combination with P[4], P[6] and P[8] commonly infect humans [13].

To reduce the burden two vaccines namely Rotarix (RV1; monovalent G1P[8]; GlaxoSmithKline Biologicals, Rixensart, Belgium) and RotaTeq (RV5; pentavalent G1, G2, G3, G4,P[8]; Merck Vaccines, Whitehouse Station, NJ, USA) were approved by FDA in 2006. Large scale vaccine trials with Rotarix and RotaTeq have shown high efficacy (∼85%) in developed countries of Europe, Australia and USA. Though efficacy is low to moderate (39–72%) in low income countries of Asia and Africa, overall reduction in disease severity as estimated by reduced hospitalization and deaths due to diarrhoea, has been documented [7], [16]–[18]. Another oral live attenuated vaccine Rotavac, which was derived from a neonatal G9P[11] human bovine reassortant strain 116E has been licensed in India recently [14], [15].

For introduction of these vaccines in national immunization programme in South Asian countries like India, robust data for estimating rotavirus disease burden was required. Several case-control or cohort studies of enteropathogens associated with childhood diarrhoea have been conducted in various countries with focus on either bacterial or viral aetiologies of diarrhoea [1], [19]–[21]. In India a large number of multistate hospital based studies of rotavirus were conducted [22]–[29]. In Eastern part of India specially in Kolkata a continuous hospital based monitoring system since 2003–2013 showed a large number of hospitalization (>40%) among children due to rotavirus diarrhoea [30]–[32]. Unfortunately hospital based studies do not provide the disease incidence rates, thus as an extension of GEMS study, burden of RVA and circulating genotypes of RVA were analyzed. Age matched healthy control children were monitored to estimate the asymptomatic infection rates in community. We also investigated whether there was any unusual strain distribution among controls and diarrhoea cases as well as to identify human animal reassortant strains in the community.

## Materials and Methods

### Study population, sample collection, and processing

The present study was an extension of Global Enteric Multicenter Study (Project entitled- Diarrhoeal diseases in infants and young children in developing countries), conducted during 2008–2010 [9]. An urban slum community of Kolkata was a study site, where population of <5 years children was 13416 [56], [57]. A total number of 3582 stool samples were collected, among which, 1568 samples were obtained from children of ≤5 years of age, with moderate to severe diarrhoea, as well as from the children who were admitted in B. C. Roy Memorial Hospital for Children and Infectious Diseases Hospital in Kolkata, India. A total of 2014 samples were collected from age-sex matched healthy neighbourhood controls in same community. Inclusion criteria for symptomatic children included passing of three or more loose/watery stools within 24 hours and satisfy with at least one of the following criteria for moderate to severe diarrhoea (MSD): 1. Sunken eyes (confirmed by parent/caretaker) as more than normal; 2. Loss of skin turgor defined as an abdominal skin pinch with slow or very slow (>2 seconds) recoil; 3. Intravenous hydration administered or prescribed; 4. Hospitalization with diarrhoea or dysentery. In asymptomatic control group, children without diarrhoea for the past 7 days were enrolled within maximum of 7 days of index case enrolment [56]. Main objective of this case-control study was, to determine the attributable fraction of diarrhoea due to specific pathogen and then estimated disease burden through Demographic Surveillance System (DSS) in community [9]. The detailed methodology has been described in published article [56]. Stool samples were stored at −70°C for further study.

### Ethics Statement

Written informed consent was taken from the parents of the children for participation in this study. Potential controls were randomly selected from the population database and matched to the case by age (±2 months for cases 0–11 months and 11–23 months, and ±4 months for cases 23–59 months), gender, and residence (same or nearby neighbourhood as the case). The study was approved by the Institutional Ethical Committee, National Institute of Cholera and Enteric Diseases, (Reference No. C-48/2008 T&E). This study involved minimal risk to the patients, and did not adversely affect the rights or welfare of the patients.

The stool samples were screened for rotavirus using an enzyme-linked immunoassay (EIA) detecting the VP6 antigen as per the manufacturer’s instructions (ELISA ProSpecT Rotavirus kit, Oxoid, Basingstoke, UK) [9].

### Viral RNA extraction and genotyping

From the ELISA positive samples, rotavirus double-stranded RNA was extracted from feces by using an automated DNA/RNA extractor (EasyMag, bioMèrieux, Marcy l’Etoile, France). Complementary DNA was synthesized from the extracted viral RNA through reverse transcription in the presence of random hexamers. G and P typing was performed using VP7- and VP4-specific multiplex semi-nested RT-PCRs as described previously [33]. PCR products were purified with a QIAquick PCR purification kit (QiagenGmbH, Hilden, Germany).

### Nucleotide sequence and phylogenetic analysis

Nucleotide sequencing was carried out using the ABI Prism Big Dye Terminator Cycle Sequencing Ready Reaction Kit v3.1 (Applied Biosystems, Foster City, California, USA) in an ABI Prism 3730 Genetic Analyzer (PE Applied Biosystems, Foster City, California, USA) as described previously [28]. Nucleotide and protein sequence BLAST search was performed using the National Centre for Biotechnology Information (NCBI, National Institutes of Health, Bethesda, MD) Basic Local Alignment Search Tool (BLAST) server on GenBank database release 143.0 [34]. Pairwise sequence alignments were performed using LALIGN software (EMBnet, Swiss Institute of Bioinformatics, Switzerland), and multiple alignments were done with DDBJ software and CLUSTAL W. Amino acid sequences were deduced using the TRANSEQ software (Transeq Nucleotide to Protein Sequence Conversion Tool, EMBL-EBI, Cambridgeshire, UK). Phylogenetic tree was constructed using the MEGA (Molecular Evolutionary Genetics Analysis) program, version 6. Genetic distances were calculated using the Maximum likelihood method (500 bootstrap replicates) using the MEGA 6 program. According to model testing results, Kimura2 parameter model was selected to construct the phylogenetic dendrograms. Lineage designation for phylogenetic dendrograms of G1, G2, G9 and G12 strains were based on those reported in previous studies [35]–[38]. Partial nucleotide sequences of VP4 gene and complete nucleotide sequences of VP7 gene of the strains detected during this study were submitted to the GenBank database under the accession numbers: KM008633–KM008642 (G1 strains); KM008643–KM008652 (G2 strains); KM008653–KM008655 (G4 strains); KM008657–KM008666 (G9 strains); KM008667–KM008675 (G12 strains); KM008676–KM008682 (P[4] strains); KM008683–KM008690 (P[6] strains); KM008691–KM008699 (P[8] strains).

## Results

### Detection of rotavirus among enrolled children in Kolkata

**Figure 1:**
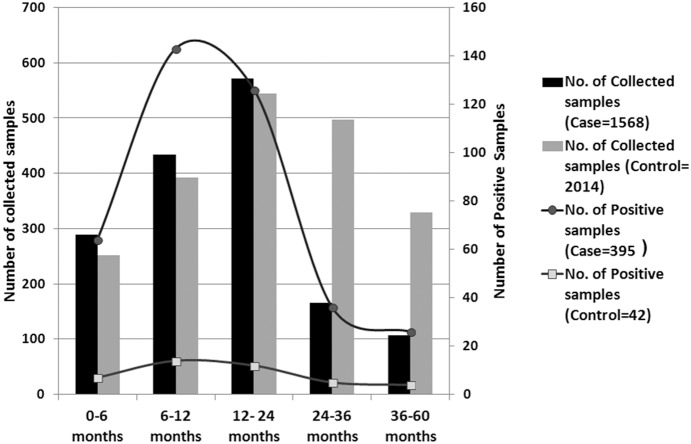
Age distribution. Age –wise distribution of rotavirus-positive Case and Control children (0–5 years) against total collected samples during January 2008 through December 2010.

**Figure 2:**
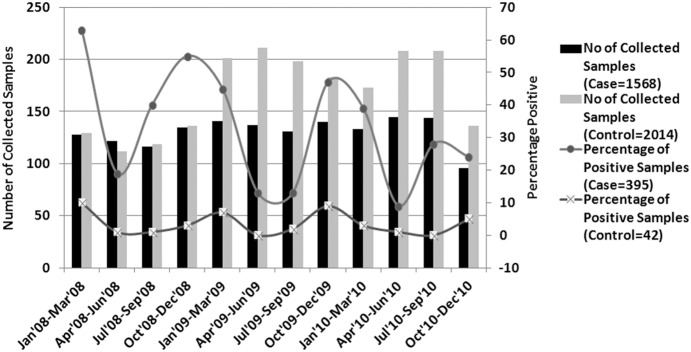
Seasonality of rotavirus in Kolkata. Seasonal distribution of the rotavirus positivity against total collected samples in Case and Control children in an urban slum community in Kolkata, India, during January 2008 through December 2010.

Among 1568 children (age, ≤5 years) with diarrhoea, 395 (25.2%), and among 2014 asymptomatic children, 42 (2%) were rotavirus ELISA positive. Highest numbers of rotavirus were found in 6–24 months age group children in both case and control population (Figure 1). Detection of rotavirus infection was highest during October to March (Figure 2). G and P typing was done for all 437 samples using multiplex RT-PCR with VP7- and VP4- type specific primers. Among 437 of rotavirus positive samples, only 9 rotavirus positive strains remained untypable for both G and P types. The globally common strain G1P[8] was identified as the most common strain (32%) followed by G9P[8] (16.9%), G2P[4] (13.5%) and G9P[4] (10.75%) (Table 1). G12 strains with combinations of P[4], P[6] and P[8] comprised 11.9% of total positive strains. The rest (<10%) were rare and uncommon strains like G1P[4], G1P[6], G2P[8] and animal-like strains G4P[6], G6P[14] and G11P[25]. Among G types, G1 (37.8%) and G9 (29.5%) strains were most prevalent and comprised 67% of the total strains (Table 1). Among eight G9 and one G12 strain, the P type could not be determined. The 42 RVA positive samples from healthy children revealed similar genotypes like G1, G2 and G9. Most of the uncommon and animal-like rotaviruses were detected from diarrhoea patients, only one unusual strain G6P[14] was detected from an asymptomatic control sample (Table 2).

**Table 1:** Distribution of rotavirus strains genotype at Kolkata, India, during 2008–2010.

P Type	P[4]	P[6]	P[8]	P[14]	P[25]	P-NT	Total
**G Type**							
**G1**	10 (2.3)	15 (3.4)	140 (32)	0	0	0	165 (37.8)
**G2**	59 (13.5)	0	18 (4.1)	0	0	0	77 (17.6)
**G4**	0	3 (0.68)	0	0	0	0	3 (0.68)
**G6**	0	0	0	1 (0.22)	0	0	1 (0.22)
**G9**	47 (10.75)	0	74 (16.9)	0	0	8 (1.8)	129 (29.5)
**G11**	0	0	0	0	1 (0.22)	0	1 (0.22)
**G12**	1 (0.22)	26 (5.95)	24 (4.49)	0	0	1 (0.22)	52 (11.9)
**G-NT**	0	0	0	0	0	9 (2.05)	9 (2.05)
**Total**	117 (26.8)	44 (10.06)	256 (58.6)	1 (0.22)	1 (0.22)	18 (4.1)	437 (100)

**Table 2:** Age and type wise distribution of Rotavirus positive Case and Control stool samples (During the year 2008 through 2010).

AgeGroup	No. OfCollectedsamples(Case)	No. of Positivesamples (Case)	No. ofCollectedsamples(Control)	No. of PositiveSamples(Control)
0–6months	**289**	**64 (22.1%)** G1P[8] = 21; G1P[6] = 5; G2P[4] = 20; G2P[8] = 5; G4P[6] = 1; G9P[4] = 3; G9P[8] = 5; G12P[6] = 3; G12P[8] = 1	**252**	**7 (2.8%)** G1P[8] = 4; G9P[4] = 1; G9P[8] = 2
6–12months	**434**	**143 (32.9%)** G1P[8] = 32; G1P[4] = 4; G1P[6] = 4; G2P[4] = 15; G2P[8] = 6; G4P[6] = 1; G9P[4] = 21; G9P[8] = 29; G12P[4] = 1; G12P[6] = 15; G12P[8] = 10; G(NT)P[NT] = 5	**392**	**14 (3.5%)** G1P[8] = 8; G9P[4] = 3; G6P[14] = 1; G12P[6] = 1; G12P[NT] = 1
12–24months	**572**	**126 (22%)** G1P[8] = 50; G1P[6] = 3; G1P[4] = 2; G2P[4] = 18; G2P[8] = 4; G4P[6] = 1; G9P[4] = 13; G9P[8] = 16; G9P[NT] = 3; G12P[6] = 7; G12P[8] = 5; G(NT)P[NT] = 4	**544**	**12 (2.2%)** G1P[8] = 4; G1P[6] = 1; G2P[4] = 1; G2P[8] = 2; G9P[4] = 2; G9P[8] = 2
24–36months	**166**	**36 (21.7%)** G1P[8] = 7; G1P[6] = 2; G1P[4] = 3; G2P[4] = 5; G9P[8] = 5; G9P[NT] = 5; G11P[25] = 1; G12P[8] = 8	**497**	**5 (1%)** G1P[8] = 2; G9P[4] = 1; G9P[8] = 2
36–60months	**107**	**26 (24.2%)** G1P[8] = 10; G2P[8] = 1; G9P[4] = 3; G9P[8] = 12	**329**	**4 (1.2%)** G1P[8] = 2; G1P[4] = 1; G9P[8] = 1
Total	**1568**	**395 (25.2%)**	**2014**	**42 (2%)**
0–6months	**289**	**64 (22.1%)** G1P[8] = 21; G1P[6] = 5; G2P[4] = 20; G2P[8] = 5; G4P[6] = 1; G9P[4] = 3; G9P[8] = 5; G12P[6] = 3; G12P[8] = 1	**252**	**7 (2.8%)** G1P[8] = 4; G9P[4] = 1; G9P[8] = 2

### Analysis of VP7 gene

The VP7 genes of 44 randomly selected rotavirus strains were analyzed following sequencing of the complete ORF (nt 49–nt 1026). Based on nucleotide sequences, phylogenetic dendograms for representative G1 (10/165), G9 (10/129), G2 (10/77), G4 (3/3), G12 (9/52), G6 (1/1) and G11 (1/1) rotaviruses were analyzed with other previously reported representative strains, belonging to the individual G types.

### Analysis of G1 strains

**Figure 3:**
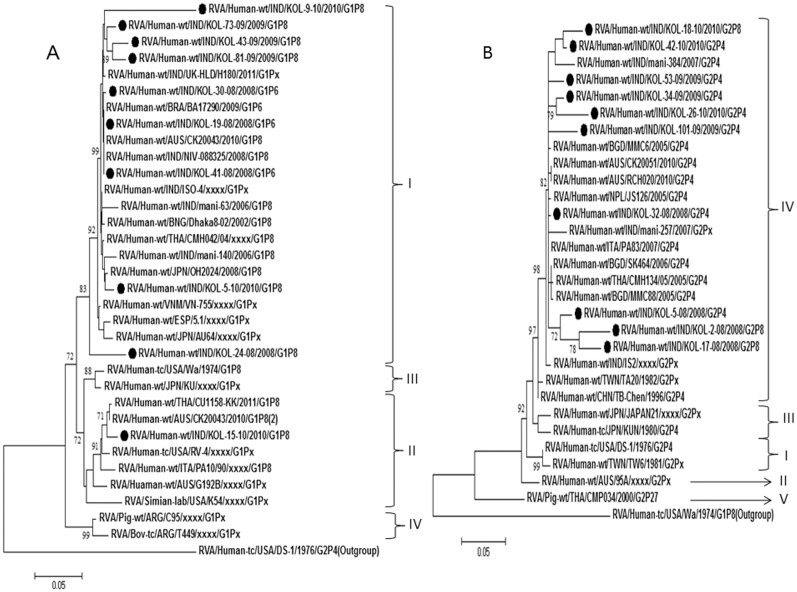
A and B. Phylogenetic trees of the G1 and G2 strains of Kolkata. Phylogenetic trees constructed from the nucleotide sequences of VP7 genes of A. G1; B. G2 strains of Kolkata, isolated during January 2008 through December 2010, with other representative G1 and G2 strains respectively. A. One Kolkata G1 strain showed its relatedness to lineage II G1 strains and the rest G1 strains clustered with lineage I G1 strains. B. All Kolkata G2 strains clustered with lineage IV G2 strains. Scale bar, 0.05 substitutions per nucleotide. Bootstrap values less than 70% are not shown.

G1 rotaviruses exhibited 97–99% nucleotide identities with strains from Australia, Thailand and Japan as well as the previously reported Indian strains (Table 3). Phylogenetic analysis revealed clustering of G1/Kolkata strains with the common G1 strains within lineage I. One strain (Kol-15-10) out of 10 clustered in lineage II (Figure 3A).

**Table 3:** Diversity of rotavirus genotype and their origin at Kolkata, India, during 2008–2010.

Types	Homology	Origin
**Common Types**		
**G1 (n = 165)**	OH2024 (Japan), CMH042 (Thailand), NIV-088325 (India), BA17290 (Brazil), CK20043 (Australia)	Human
**G2 (n = 77)**	MMC6 (Bangladesh), CMH134 (Thailand), RCH020 (Australia), CK20051 (Australia), PA83/2007 (Italy)	Human
**G9 (n = 129)**	ISO95 (India), CAU202 (South Korea), 2371WC (South Africa), AV21 (Italy)	Human
**G12 (n = 52)**	Mani-485 (India), ISO-16 (India), Dhaka12 (Bangladesh), GER172 (Germany), ISO27 (India), VU08-09-6 (USA)	Human
**P** [4] ** (n = 117)**	CAU209-KK (Thailand), 01076 (Russia), CMH028 (Thailand), Omsk08-464 (Russia), CK20043 (Australia)	Human
**P** [8] ** (n = 256)**	GRAVP420 (India), VU08-09-39 (Vanderbilt), CAU202 (South Korea), MRC-DPRU1417/2009 (Cameroon)	Human
**Uncommon Types**		
**G4 (n = 3)**	HeN4 (China)	Porcine
**G6 (n = 1)**	RUBV319 (India)	Bovine
**G11 (n = 1)**	KTM368 (Nepal), YM (Mexico)	Human, Porcine
**P** [6] ** (n = 44)**	GER172 (Germany), SK277 (Japan), GUB88 (Japan)	Human, Porcine
**P** [14] ** (n = 1)**	Hun5 (Hungary)	Human
**P** [25] ** (n = 1)**	Dhaka6 (Bangladesh), KTM368 (Nepal)	Human

### Analysis of G2 strains

Comparative analysis of 7 G2 strains showed high sequence identity (98.3–99.6%) with G2 strains of Bangladesh, Thailand, Australia and Italy within lineage IV. But 3 G2 strains, Kol-2-08, Kol-5-08 and Kol-17-08, showed relatively lower nucleotide identity (92–95%) with the lineage IV strains. But according to phylogenetic analysis, all Kolkata G2 strains remain within lineage IV (Figure 3B).

### Analysis of G9 strains

**Figure 4:**
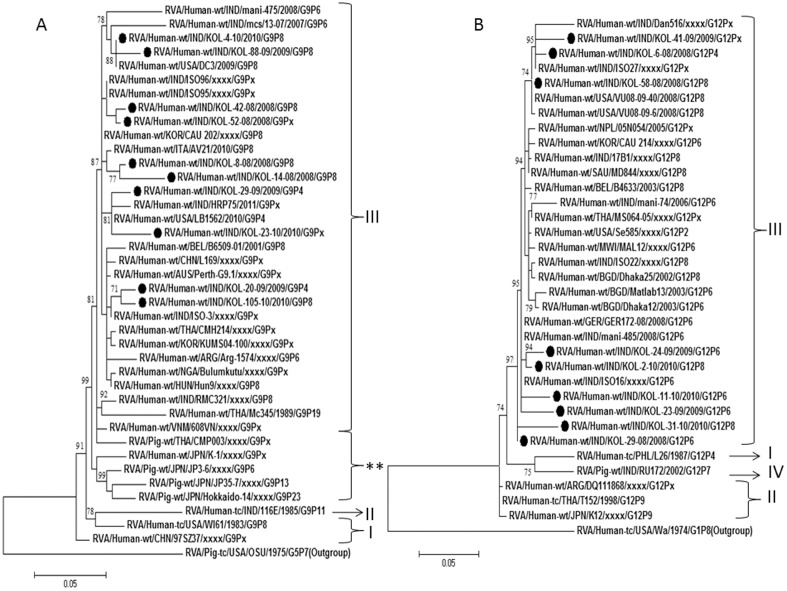
A and B. Phylogenetic trees of the G9 and G12 strains of Kolkata. Phylogenetic trees constructed from the nucleotide sequences of VP7 genes of A. G9; B. G12 strains of Kolkata, isolated during January 2008 through December 2010, with other representative G9 and G12 strains respectively. A. All Kolkata G9 strains showed relatedness to lineage III G9 strains. (**Sub cluster of Japanese and Chinese rotavirus strains). B. All Kolkata G12 strains clustered with lineage III G12 strains. Scale bar, 0.05 substitutions per nucleotide. Bootstrap values less than 70% are not shown.

G9 was the second highest genotype observed during this study. Most of the G9 strains were found with P[8] and P[4] specificity, however a few (1.8%) could not be typed for VP4 gene. Nucleotide and amino acid blast analysis as well as phylogenetic analysis revealed close clustering of Kolkata G9 strains with lineage III G9 strains reported from India, South Korea, Italy and South Africa (Table 3; Figure 4A).

### Analysis of G12 strains

G12 strains were found in combination with P[4], P[6] and P[8]. Following nucleotide analysis, all G12 rotaviruses shared maximum identity (≈97%) with G12 strains reported from Bangladesh (Dhaka 12-03), India (mani-485) and Germany (GER172-08) (Table 3). Phylogenetic analysis revealed clustering of current Kolkata G12 strains with lineage III G12 strains (Figure 4B).

### Analysis of G4 strains

**Figure 5:**
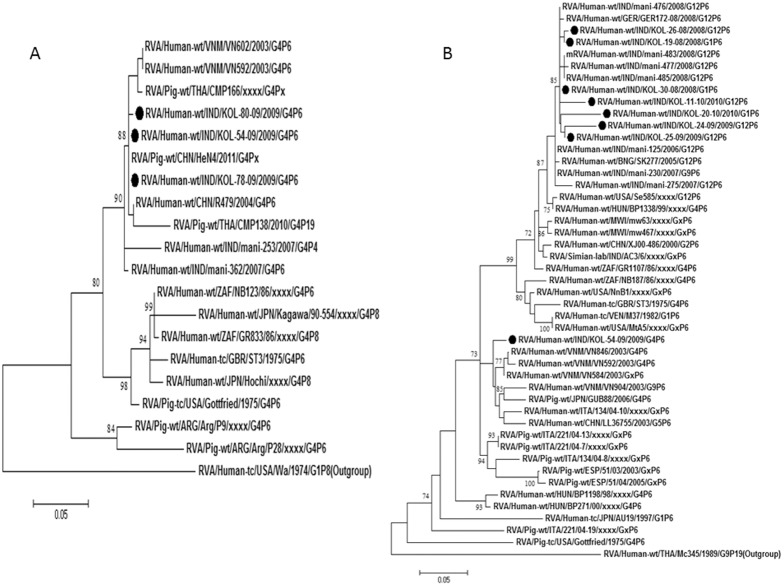
A and B. Phylogenetic trees of the G4 and P[6] strains of Kolkata. Phylogenetic trees constructed from the nucleotide sequences of VP7 genes of A. G4; and VP4 genes of B. P[6] strains of Kolkata, isolated during January 2008 through December 2010, with other representative G4 and P[6] strains respectively. All G4 strains indicated its genetic relatedness with the porcine or porcine-like human G4 strains. Scale bar, 0.05 substitutions per nucleotide. Bootstrap values less than 70% are not shown.

Only three G4 strains Kol-54-10, Kol-78-10 and Kol-80-10 were identified during the study with P[6] specificity. Preliminary BLAST analysis of VP7 gene of these G4 strains revealed genetic relatedness to porcine G4 strains of China, HeN4 with nucleotide identity of 98% (Table 3). In the phylogenetic dendrogram, also these strains clustered with the porcine strains of China along with porcine-like human G4 strains of India and Vietnam (Figure 5A).

### Analysis of G6 and G11 strain

Only one G6 strain RVA/Human-wt/IND/N-1/2009/G6P[14] and one G11 strain RVA/Human-wt/IND/N-38/2009/G11P[25] were identified during this study. The G6 strain N-1/2009, shares 95% nucleotide identity and 97% amino acid homology with previously reported bovine strain RUBV319 from India [39]. N-1/2009 was found to be in the same cluster with Indian bovine strains and with a caprine strain GO34 from Bangladesh in phylogenetic dendrogram. The G11 strain N-38/2009 showed 98% sequence identity with a porcine-like human strain CRI10795 previously reported from India and 91% nucleotide similarity with a Mexican porcine strain YM. N-38/2009 clustered with porcine-like human G11 strains from Bangladesh, Nepal, India and South Korea [39], all of which were originated from porcine G11 strain YM.

### Analysis of VP4 gene

Partial VP4 gene (nt 1–nt 881; VP8* complete ORF 247 aa) sequences of the 26 strains (7 genotype P[4], 8 genotype P[6], and 9 genotype P[8], 1 genotype P[14], 1 genotype P[25] strains) were analyzed.

### Analysis of P[4] and P[8] strains

**Figure 6:**
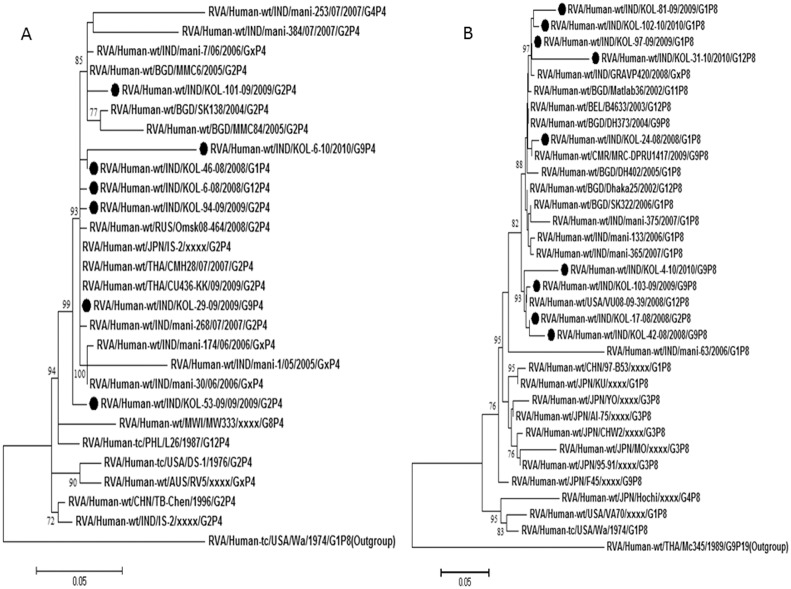
A and B. Phylogenetic trees of the P[4] and P[8] strains of Kolkata. Phylogenetic trees constructed from the nucleotide sequences of VP4 genes of A. P[4]; and B. P[8] strains of Kolkata, isolated during January 2008 through December 2010, with other representative P[4] and P[8] strains respectively. Scale bar, 0.05 substitutions per nucleotide. Bootstrap values less than 70% are not shown.

P[4] strains were detected with G1, G2, G9 and G12 genotypes, though majority of P[4] (24%) genotype was associated with G2 and G9. Nucleotide and amino acid sequence analysis revealed that P[4] genotype associated with G2 strains, were more than 97% homologous with common human strains reported from Thailand and India (Table 3). Other P[4] strains, shared of ≈96% nucleotide identity with the human P[4] strains of Japan, Bangladesh, Russia and worldwide (Table 3; Figure 6A). The P[8] rotaviruses commonly associated with G1 genotype, whereas few associated with G9, G2 and G12 also. P[8] associated with G1 strains and one G12 strain, Kol-31-10 exhibited nucleotide identity (96–99%) with RVA strains of India and Bangladesh. Other P[8], associated with G2, G9 and G12 shared 99% nucleotide identity with a G12P[8] strain from Vanderbilt, USA (Table 3; Figure 6B).

### Analysis of P[6] strains

Eight strains with P[6] genotypes were further analyzed. P[6] strain associated with G4 were 95% nucleotide homolog to the VP4 gene of a Japanese porcine strain GUB88. Other P[6] strains associated with G1 and G12 clustered with other recently emerging porcine-like human P[6] strains from India, Germany, and Japanese porcine strains as shown in phylogenetic tree (Figure 5B).

### Analysis of P[14] and P[25] strain

P[14] and P[25] genotypes are very rare. A few of P[14] genotypes are reported in bovine/bovine-like human strains to date from several parts of Europe and India [40]–[42]. P[25] genotypes are reported previously in Bangladesh, Nepal and India [43], [44]. As described previously, P[14] strain detected in this study originated from a bovine strain of India and P[25] strain was found to share its homology with porcine-like human strain KTM368 reported from Nepal [39].

## Discussion

Deaths due to rotavirus infection are uncommon in industrialized countries, but rotavirus infection remains an important cause of mortality among young children in the developing countries [45]–[47]. Due to huge genetic diversity, interspecies transmission, genetic reassortment and recombination, vaccines have not been successful in controlling the infection though vaccination has significant effect on reducing disease severity and hospitalization rates [16].

In developing countries like India where diarrhoea related mortality is high continuous monitoring of the circulating genotypes in the community is required, prior to implementation and evaluation of candidate vaccines. Community based studies are thus important to assess the impact of immunization on the prevalence of common genotypes, emergence of strains that escape immunity and evolution of the genes of wild type rotaviruses as a consequence of immune selective pressure. Inclusion of asymptomatic control children to this study helped to figure out the exact burden and risk factors of rotavirus infection in community, because asymptomatic infections are potential source of dispersal of infection in environment.

During this study, in the year 2008 through 2010, in an urban slum community in Kolkata, India, 25.2% of the children with severe diarrhoea, were detected positive for rotavirus, and 2% of the asymptomatic children were rotavirus positive [9]. As expected percent positivity of rotavirus in community settings (25.2%) is lower compared to ∼40%±5% positivity observed in hospital based studies [26], [28], [30], [31], [48]. Consistent with previously reported seasonality of RVA infection in India, in this study maximum positivity was observed during October to March (Figure 2) when average temperature remained 21°–23°C [26], [28], [32]. Similarly age wise distribution revealed maximum rotavirus positive children of 6–24 months old (Figure 1). This is probably due to start of weaning period from 6 months age when child is introduced to semi solid food and infant ready food mixes thus increasing exposure to water and other environmental source of contaminations. We could not found any distinct variation in strain distribution within case and control children. Common strains like G1, G2, G9 and G12 were found within both case and control children (Table 2).

Recent reviews revealed G1P[8], G2P[4], G3P[8] and G4P[8] as the globally important combinations of rotavirus strains detected worldwide [19]. In Kolkata, previous studies during 2003–2007, showed that, in the Eastern part of India, G1 (>50%) and G2 strains (∼23–33%) were dominant, whereas G9 (2–10%) and G12 (8–17%) strains occurred at varied frequency over the period of time (2003–2007) [30], [31]. The community based study (2008–2010) showed increasing trend of G9 strains (29.5%) whereas G1 strains (37.8%) were predominant (Table 1). Interestingly the follow up hospital based study in Kolkata during 2011–2013, revealed overall reduction of G1 strains (∼16%) and significant increase in G9 strains (∼40%) and G2 (∼36%) strains [32]. This indicates it was a gradual process of replacement of G1 strains by G9 strains. One of the reasons for the emergence of G9 strains worldwide was that the rare genotypes like the G12 or G9 might escape recognition by the host immune system which recognizes the common G1–G4 genotypes. In addition if complete cross protection is not achieved by current vaccines, RotaTeq (G1–G4 P[8]) and Rotarix (G1P[8]) a selective increase in the prevalence of G9 or other emerging genotypes is possible even though cross protection to other genotypes has been documented [16]. However, in India the increase in G9 strains cannot be attributed to vaccines yet as RVA vaccines have still not been introduced in national immunization program. Due to high incidence of G9 strains in India, efficacy studies on newly licensed G9 based vaccine 116E are being evaluated [15]. Sequence analysis revealed all G9 strains to be similar to lineage III strains reported previously from Asia, Africa and Europe [31], [32], [53]–[55]. During previous studies in India G9 strains were commonly observed in combination with P[6] or P[8] [28], [31], but during this period almost 10.75% strains belong to G9P[4] genotype. As G9 is normally associated with Wa-like genotype strains and P[4] belongs to Ds-1-like genotype, thus increased occurrence of G9P[4] may be a result of intergenogroup reassortment [58]. Further full genome analysis of these strains is required to understand origin of these G9P[4] strains.

G12 strains have been reported from India, Bangladesh, Brazil, Spain, USA and other countries with varying frequency in association with either P[6] or P[8] genotypes [26], [49]–[52]. G12 strains detected in current study belong to lineage III (Figure 4B) consistent with the previously reported G12 strains worldwide. Other strains like G4, G12, G6, G11 in combination with P[6], P[14], P[25] comprised ∼15% of all genotypes (Table 1). Consistent with previous surveillance data from Kolkata, G3 strains were not observed and G4 strains, which were all derived from animal origin (Figure 5A), circulated at very low frequency (∼1%).

Systemic case-control study confirms co-circulation of all major genotypes G1, G2, G9, G12, detection of unusual strains, and zoonotic transmissions reflecting the complex epidemiology of group A rotaviruses in India. Such variations may be facilitated by high density population, poor unhygienic conditions, and lack of safe drinking water. Preventive strategies targeting first 2 years of life may accelerate effectiveness in disease control. Continuous longitudinal surveillance programs both before and after introduction of rotavirus vaccine will shed light on the long term efficacy of rotavirus vaccines in India.
